# ABRF 2026 Awards

**DOI:** 10.7171/001c.162638

**Published:** 2026-06-09

**Authors:** Sheenah Mische, Richard Cole

**Affiliations:** 1 Pathology New York University; 2 Microscopy New York State Department of Health

**Keywords:** shared resources, awards, scientific professional society, biomolecular technology

## Abstract

The Association of Biomolecular Resource Facilities (ABRF) presents several awards annually to recognize exceptional contributions to pioneering research, biomolecular technologies, scientific excellence, career mentorship, leadership, and outstanding advocacy for inclusive science. These awards were presented during the Annual Meeting held in Pittsburgh. The top honors recognized significant scientific breakthroughs, diversity advocacy, and outstanding community contributions in biomolecular research.

## The 2026 Association of Biomolecular Facilities (ABRF) Awards

The Association of Biomolecular Resource Facilities (ABRF) 2026 Awards were presented at the Annual Meeting in Pittsburgh, PA (March 28–31). The event honored leading professionals for their contributions to technology, diversity, and the broader research community.

The 2026 award recipients are as follows.

## ABRF Award for Outstanding Contributions to Biomolecular Technologies

**Figure:**
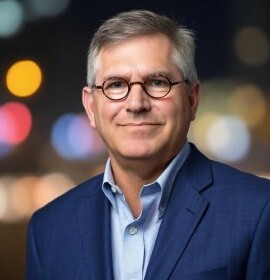


This ABRF Award recognizes those pioneers responsible for developing powerful new technologies and tools that form the foundation upon which the modern biomolecular research enterprise thrives, or those who have significantly improved/advanced the impact of shared research facilities.

John R. Yates, PhD, Ernest W. Hahn Professor in the Departments of Molecular Medicine and Neurobiology at The Scripps Research Institute, was selected as the recipient of the 2025 ABRF Award for his transformative work in proteomics and the SEQUEST algorithm.

## The Spencer Shorte Legacy Award (SSLA)

Spencer Shorte was widely recognized for his exceptional scientific contributions, but the **Spencer Shorte Legacy Award** honors more than professional achievement; it celebrates the profound humanity that defined his life. A gifted scientist and multidisciplinary investigator, Spencer merged technologies to advance discovery across decades and continents.

Yet, Spencer is best remembered for his extraordinary character. His humility fostered open collaboration, his community focus inspired those around him, and his selflessness consistently prioritized others’ needs. The warmth he extended to everyone he met left a lasting impact, and his legacy lives on in the colleagues he elevated and nurtured with grace. This award serves as a living tribute to Spencer’s memory by honoring individuals who similarly embody these core values: those researchers who are recognized not for a list of publications, but for their integrity, kindness, and dedication to uplifting the scientific community.

ABRF is proud to name **Dr. Kevin Eliceiri** as the **2026 inaugural recipient of the Spencer Shorte Legacy Award**. Dr. Eliceiri is the Walter H. Helmerich Research Chair and Professor of Medical Physics and Biomedical Engineering at the University of Wisconsin–Madison.

In the spirit of Spencer’s commitment to community building, the award will support Dr. Eliceiri’s establishment of the Illuminating People Program (IPP). This innovative matchmaking platform is designed to recognize and foster the “bidirectional intellectual spark” inherent in effective mentorship.

**Figure:**
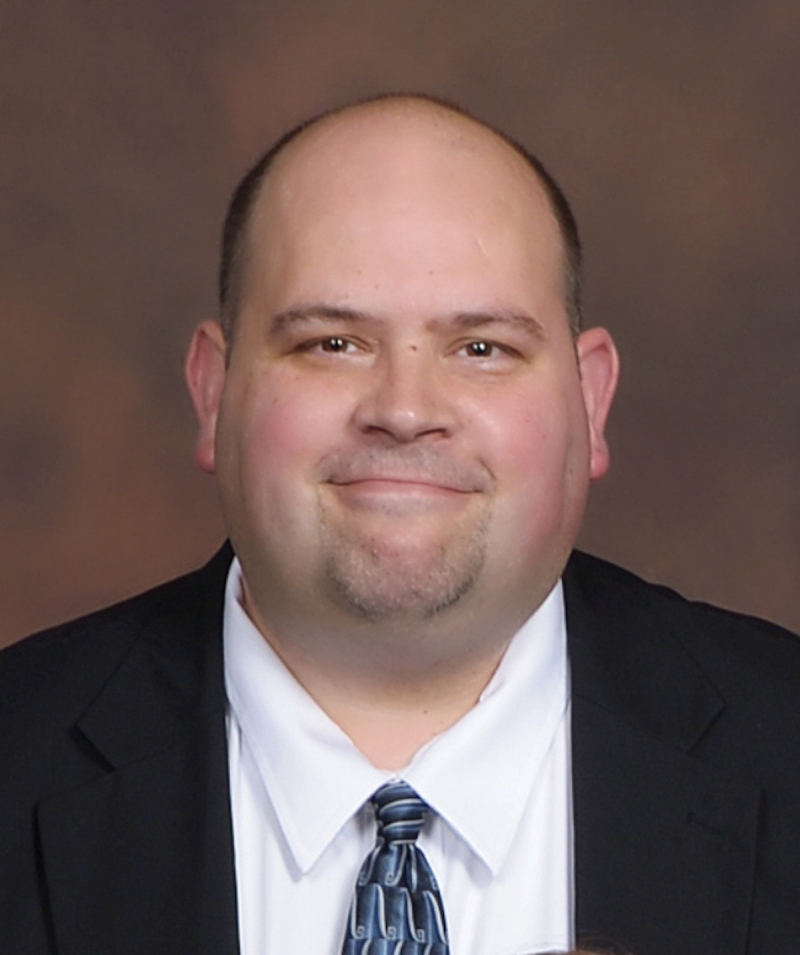


The Illuminating People Program (IPP) will:

- Bridge connection gaps by match experienced researchers with early-career scientists, specifically targeting candidates who lack existing relationships with potential mentors.
- Reward professional growth through the IPP Fellows program where mentees and advice-seekers will receive recognition and support as they invest in their professional goals.
- Launch a sustainable pilot with the initial two-year pilot supporting a cohort of at least 20 students, who will be selected through a need-based application and review process.
- Build a long-term legacy through ongoing outreach that will highlight the mutual benefits of the program and seek to secure endowed funding to ensure the IPP remains a permanent resource for the community.

By fostering these vital human connections, Dr. Eliceiri continues the work that Spencer Shorte championed: ensuring that kindness, collaboration, and mentorship remain the heartbeat of scientific progress.

The presentation at the ABRF annual meeting describing the work recognized by the ***ABRFSSL Award*** includes travel, meals, and accommodation expenses to attend the ABRF Annual Meeting and various recognition events during the meeting, and it also includes funds ($15,000) toward a recipient-led program that is focused on community building.

## The ABRF Diversity, Equity, Inclusion, and Belonging (DEIB) Award

Established in 2020, the **ABRF Diversity, Equity, Inclusion, and Belonging (DEIB) Award** reflects the association’s steadfast commitment to social justice. The award was created to honor individuals, groups, or organizations whose work fosters a more diverse, inclusive, and equitable scientific community. In its selection process, the committee prioritizes nominees whose efforts directly impact the ABRF community and embody the association’s mission to act proactively and unapologetically in the pursuit of inclusion and justice.

**ABRF** is proud to announce **Dr. Mariana De Niz** as the recipient of the **2026 ABRF DEIB Award**. This honor recognizes her transformative leadership and unwavering advocacy for equity across the global scientific landscape.

**Figure:**
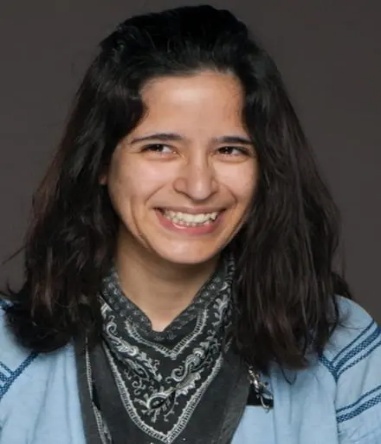


Dr. De Niz, a Research Assistant Professor of Cell and Developmental Biology and the Global Engagement Manager and Advanced Microscopy Specialist at the **Northwestern University Center for Advanced Microscopy**, has dedicated her career to democratizing access to scientific technology.

Her impact spans several key international leadership roles and initiatives:

- Breaking Barriers: As an ambassador for the **Royal Microscopy Society** and leader of the Outreach and Integration working group for **Latin America Bioimaging (LABI)**, she works to dismantle the silos between the scientific community and the general public.
- Elevating Visibility: Through *FocalPlane*, she has highlighted the achievements of Latin American microscopists, fostering local and international collaborations. Alongside **Dr. Constadina Arvanitis** (recipient of the 2026 ABRF Alan Smith Mentor Award), she cofounded the blog *“Towards Global Access”* to spotlight worldwide efforts in scientific equity.
- Community Advocacy: As Chair of the **Bioimaging North America (BINA)** Community Engagement working group, Dr. De Niz has led initiatives to recognize the contributions of underrepresented Latin American, Indigenous, African American, and Asian American communities.

Dr. De Niz is a vocal advocate for scientists with disabilities and has pioneered panels addressing mental health from the perspectives of both individual researchers and core facility leaders. Recognizing that language can be a barrier to knowledge, she contributed to the translation of essential resources, including Beth Cimini’s *Bioimaging Guide* and Peter Bankhead’s *Bioimage Book*. Moreover, her selection for the **PiTCH program**, led by the **Africa Microscopy Initiative**, underscores her commitment to working with African partners and international mentors to facilitate knowledge exchange and strengthen global microscopy networks.

Dr. Mariana De Niz’s passionate leadership across these diverse initiatives embodies the spirit of the DEIB Award and drives the scientific community toward a more inclusive and accessible future.

## The Alan Smith Mentor of the Year Award

As a founding member of **ABRF**, Alan J. Smith is remembered for his profound dedication to professional mentoring and guidance. In his honor, the **Alan Smith Mentor of the Year Award** recognizes members who embody this commitment to leadership. Reviewed and selected by the ABRF Career Development Committee, this award celebrates mentors who bridge generational gaps and train the next generation of scientists, ensuring the continued innovation and excellence of shared research resources.

**Figure:**
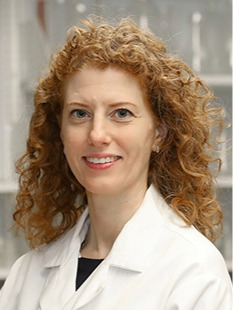


ABRF is proud to present the 2026 Alan Smith Mentor of the Year Award to **Dr. Constadina (Dina) Arvanitis** in recognition of her distinguished contributions to the development of next-generation scientists.

As a Research Associate Professor and Director of the **Center for Advanced Microscopy at Northwestern University’s Feinberg School of Medicine**, Dr. Arvanitis views teaching and advising as the essential cornerstones of scientific advancement. She embodies the core qualities of an exemplary mentor—empathy, patience, and adaptability—and maintains a deep commitment to the holistic professional and personal growth of her students and peers alike.

Her impact extends beyond her home institution through her significant collaborative outreach. In partnership with her colleague **Dr. Mariana De Niz** (the 2026 ABRF DEIB Award recipient), Dr. Arvanitis cofounded the blog *“Towards Global Access”* on the *FocalPlane* platform. This initiative highlights international efforts to democratize scientific knowledge and technology, further demonstrating her commitment to global mentorship.

Dr. Arvanitis’s leadership continues to inspire the ABRF community and foster an environment where the next generation of researchers can thrive.

## The ABRF Robert A. Welch Award

**Figure:**
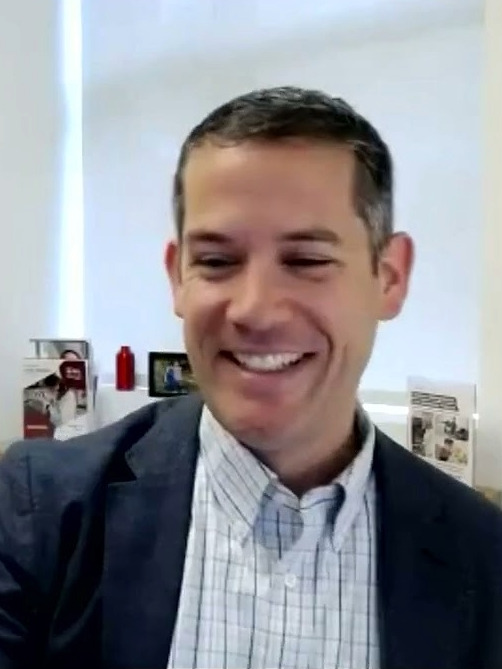


The **Robert A. Welch Outstanding Research Group or Committee Member of the Year Award** celebrates the significant contributions of individuals within ABRF’s research groups and committees. Established in 2008 and later renamed in memory of Robert A. Welch, this prestigious award honors the dedication and volunteer spirit that drive the ABRF community’s success.

ABRF is pleased to announce **Andrew Vinard** as the recipient of the **2026 Robert A. Welch Award**. Andrew’s extensive history of service and leadership has left an indelible mark on the Association’s operations and strategic vision.

Andrew has contributed to a wide array of ABRF initiatives, demonstrating a remarkable commitment to expanding shared research resources. Notable highlights of his service include:

- Executive leadership by leading the **2023 ABRF Annual Meeting Program Committee**.
- Regional & specialized service through being a leadership member of the **Northeast Regional Life Sciences Core Directors (NERLSCD)** organizing committee and the **Core Administrators’ Network Coordinating Committee (CANCC)**.
- Strategic vision by chairing the **ABRF Strategy Task Force on Communicating the Value and Impact of Shared Research Resources**, a critical initiative to articulate the essential role of shared research resources (SRRs) in modern science.

The impact of Andrew’s leadership is perhaps best captured by the words of a colleague and fellow ABRF member: “Andrew’s ability to bring structure to complex discussions, coordinate diverse stakeholders, and keep the team focused has been invaluable to our collective success.” Andrew’s tireless efforts embody the spirit of the Robert A. Welch Award, ensuring that ABRF remains a robust and forward-thinking organization for all its members.

## The Journal of Biomolecular Techniques (JBT) Outstanding Manuscript Award

The **Journal of Biomolecular Techniques (JBT) Outstanding Manuscript Award** is an annual honor recognizing the best research article published in the *Journal of Biomolecular Techniques* (JBT). The award, presented by ABRF, highlights significant contributions to biotechnology and SRR core laboratory methodologies. The JBT editorial board reviews the publication metrics and impact of each JBT article, and the winning article is recognized for its exceptional impact on the scientific community.

The 2026 JBT Outstanding Manuscript Award winner was **“Voices from the Bench: Focus Group Insights on Shared Research Resource Sustainability Amid Federal Policy Shifts”,** authored by **A.N. White, R. Ingersoll, J. Eswaraka, R. Uthamanthil, G. Roble, A.I. Chitty, N. Nikolaidis, A. Vinard, M. Kraft, and M.K. Winter.** The article, the result of a detailed survey with contributions and insights from over 220 academic and SRR leaders, outlines a roadmap for the resilience of SRRs and promotes their integration as institutional strategic partners to build a sustainable research ecosystem.

## ABRF Lightning Talk Awards

Waters Corporation sponsors prizes for the best lightning talks presented at the ABRF Annual Meeting of original research. Eligible talks focus on the latest scientific research results enabled by advanced life science technologies, methods, and software tools that facilitate applications, as well as the latest technological developments in the biotechnology field. The ABRF education committee (EdComm) reviews and selects eligible candidates for award consideration. Lead authors are invited to give a short (3-minute) presentation of their work. Each winner receives $500, complimentary ABRF membership for one year, and a reduced registration fee for the ABRF Annual Meeting.

The four 2026 researchers recognized for their presentations were **Drew Hankiewicz,** Van Andel Institute; **Christopher Nagy,** University of North Carolina, Chapel Hill; **Carole Perrot**, Johns Hopkins University; and **Aodong Qiu,** University of Pittsburgh.

## The ABRF Founder’s Award

This endowed scholarship is designed to support early-career SRR staff with leadership promise. Made possible by Ron Niece, one of ABRF’s founding members, this award enables the recipient to advance their career by engaging in and participating in the ABRF Annual Meeting and its related education programs. The 2026 ABRF Founder’s Award recognizes **Simon Goldstein** of the La Jolla Institute for Immunology for his contributions to the SRR community.

## The ABRF Outstanding Scientist Scholarships

In recognition of their significant contributions to their institutional shared resource facility or to ABRF, 12 professionals were selected by a committee of ABRF peers for the Outstanding Scientist Scholarship Award:

- **Alex Anwar**, Mayo Clinic
- **Simon Goldstein**, La Jolla Institute for Immunology*
- **Sumeet Gupta**, Whitehead Institute, MIT
- **Rochelle Kelley**, University of California, San Francisco
- **Vikas Kumar**, University of Massachusetts Chan Medical School
- **Adarsh Mayank**, University of California, Los Angeles
- **Chris Nagy**, University of North Carolina, Chapel Hill
- **Vijaya Pandey**, University of California, Los Angeles
- **Ken Quayle**, Cincinnati Children’s Hospital Medical Center
- **Shannon Rhoads**, University of North Carolina, Chapel Hill
- **MaKayla Serres**, Mayo Clinic
- **Caroline Vergette**, Ottawa Hospital Research Institute

Our congratulations to each awardee for their contributions to the SRR community.

